# Stromal Score-Based Gene Signature: A Prognostic Prediction Model for Colon Cancer

**DOI:** 10.3389/fgene.2021.655855

**Published:** 2021-05-12

**Authors:** Jing Jia, Yuhan Dai, Qing Zhang, Peiyu Tang, Qiang Fu, Guanying Xiong

**Affiliations:** ^1^Medical Center for Digestive Diseases, The Second Affiliated Hospital of Nanjing Medical University, Nanjing, China; ^2^The First School of Clinical Medicine, Nanjing Medical University, Nanjing, China; ^3^The School of Stomatology, Nanjing Medical University, Nanjing, China

**Keywords:** colon cancer, tumor microenvironment, stromal score, prediction model, prognosis, gene signature, tumor biomarkers

## Abstract

**Background:**

Growing evidence has revealed the crucial roles of stromal cells in the microenvironment of various malignant tumors. However, efficient prognostic signatures based on stromal characteristics in colon cancer have not been well-established yet. The present study aimed to construct a stromal score-based multigene prognostic prediction model for colon cancer.

**Methods:**

Stromal scores were calculated based on the expression profiles of a colon cancer cohort from TCGA database applying the ESTIMATE algorithm. Linear models were used to identify differentially expressed genes between low-score and high-score groups by limma R package. Univariate, LASSO, and multivariate Cox regression models were used successively to select the prognostic gene signature. Two independent datasets from GEO were used as external validation cohorts.

**Results:**

Low stromal score was demonstrated to be a favorable factor to the overall survival of colon cancer patients in TCGA cohort (*p* = 0.0046). Three hundred and seven stromal score-related differentially expressed genes were identified. Through univariate, LASSO, and multivariate Cox regression analyses, a gene signature consisting of LEP, NOG, and SYT3 was recognized to build a prognostic prediction model. Based on the predictive values estimated by the established integrated model, patients were divided into two groups with significantly different overall survival outcomes (*p* < 0.0001). Time-dependent Receiver operating characteristic curve analyses suggested the satisfactory predictive efficacy for the 5-year overall survival of the model (AUC value = 0.733). A nomogram with great predictive performance combining the multigene prediction model and clinicopathological factors was developed. The established model was validated in an external cohort (AUC value = 0.728). In another independent cohort, the model was verified to be of significant prognostic value for different subgroups, which was demonstrated to be especially accurate for young patients (AUC value = 0.763).

**Conclusion:**

The well-established model based on stromal score-related gene signature might serve as a promising tool for the prognostic prediction of colon cancer.

## Introduction

Colon cancer is one of the leading causes of cancer-related morbidity and mortality worldwide (Siegel et al., 2020). To date, the AJCC stage, determined according to the tumor, node, and metastasis (TNM) system, has been generally acknowledged as the most important tool for making clinical decisions and routine prognostication for colon cancer (Hu et al., 2011). However, prognostic outcomes have been reported to be quite diverse among colon cancer patients with the same TNM stages and similar clinical characteristics. This is mainly because of the high levels of heterogeneity found in colon cancer, which indicates that the current TNM stage system failed to provide enough prognostic information for colon cancer. Therefore, it is necessary to seek out other efficient prognostic factors to improve prognosis stratification and survival outcome prediction in addition to the current staging system.

The tumor microenvironment (TME) of colon cancer is composed of immune cells and stromal cells besides tumor cells, which all play vital roles in cancer initiation and development, as well as drug resistance (Straussman et al., 2012; Hui and Chen, 2015; Kobayashi et al., 2019). In recent years, growing evidence has revealed the crucial roles of stromal cells in various malignant tumors (Zhang et al., 2003; Xu et al., 2016). For colon cancer, it has been reported that tumor-associated stromal cells can support T-cell suppression by PD-L1 induction, revealing the importance of stromal cells in suppressing CD8+ antitumor immune responses of colon cancer (O’Malley et al., 2018).

ESTIMATE (Estimation of STromal and Immune cells in MAlignant Tumor tissues using Expression data) is a newly developed method that infers stromal and immune cells based on gene expression profiles of cancer tissues (Yoshihara et al., 2013). Immune score, stromal score, estimate score and tumor purity were calculated through ESTIMATE algorithm to predict the level of infiltrating immune cells and stromal cells using the expression data of specific gene signature associated with immune and stromal components of TME. Up to date, numerous researchers have taken advantage of ESTIMATE algorithm in studies involving varieties of cancers, such as glioblastoma, gastric cancer, breast cancer and prostate cancer (Priedigkeit et al., 2017; Shah et al., 2017; Jia et al., 2018; Wang et al., 2019), suggesting the effectiveness of the big-data based algorithm. However, the role of ESTIMATE algorithm in colon cancer remains to be elucidated.

In this study, the ESTIMATE algorithm was applied to calculated the immune scores, stromal scores, estimate scores and tumor purity of a series of colon cancer tissues based on their expression profiles, and the survival analyses indicated that stromal score was prognostic for colon cancer. Then, a novel gene signature based on stromal score was developed subsequently for prognostic prediction in colon cancer.

## Materials and Methods

### Data Source and Application of ESTIMATE Algorithm

The Cancer Genome Atlas (TCGA) level 3 gene expression RNA-seq data (standardized reads per kilobase per million mapped reads) of tissues from patients with colon cancer, along with corresponding clinicopathological information were downloaded from TCGA database^1^ on Oct 15, 2020. The expression profiles for tumors with “Colon” as the primary site and the disease type of “Adenocarcinomas” from a “TCGA-COAD (Colon adenocarcinoma)” project were included. Besides expression data, only patients with significant clinicopathological information such as survival information, age, gender and pathological TNM stage were included in this study. Four ESTIMATE scores: immune score, stromal score, estimate score and tumor purity were calculated from the expression matrix applying the ESTIMATE algorithm for each patient, respectively. Two independent datasets from the Gene Expression Omnibus (GEO) database, namely GSE38832 and GSE39582, were used for external validation in this study. The gene expression array profiles and clinicopathological data of GSE38832 (*n* = 122) and GSE39582 (*n* = 521) were downloaded by GEOquery R package, and the patients with survival follow-ups and essential clinicopathological information were included into the validation cohorts. Next, the Probe IDs were transferred to gene symbol by hug133plus2.db R package. The probe with the maximum mean was reversed when more than one probe had the same matched gene name. Access to the de-identified linked dataset was obtained from TCGA and GEO in accordance with the database policy. For analyses of de-identified data from the TCGA and GEO databases, institutional review board approval and informed consent were not required.

### Correlation Between Prognosis and Four ESTIMATE Scores

Overall survival (OS) was used as the primary prognosis endpoint. Based on the four ESTIMATE scores calculated for each patient, best cut-off value for each score was determined by Survminer R package (V.0.4.6) and patients were divided into high-score and low-score groups according to their corresponding best cut-off values. The survival prognosis for each group was examined by Kaplan-Meier analysis. The survival outcomes of the two groups were compared by log-rank tests.

### Identification of Differentially Expressed Genes (DEGs)

Patients in the training cohort were divided into two groups, namely the low stromal score group and the high stromal score group. Linear models were used to identify DEGs between the two groups (low-score group vs. high-score group) by limma R package (Ritchie et al., 2015). A *p*-value < 0.0001 combined with a simultaneously absolute value of log2 [fold-change (FC)] > 1 was set as the threshold for DEG identification. Genes downregulated in the low stromal score group compared with the high stromal score group were considered as “downregulated DEGs” and those upregulated in the low stromal score group were considered as “upregulated DEGs.” The DEGs reached the threshold we set were presented on a volcano plot. Expression patterns of significant DEGs were visualized on a heatmap with unsupervised hierarchical clustering analysis.

### Gene Ontology and Kyoto Encyclopedia of Genes and Genomes Pathway Enrichment Analyses

Enrichment analyses of Gene Ontology (GO) and Kyoto Encyclopedia of Genes and Genomes (KEGG) pathway for identified DEGs were performed using clusterProfiler R package. An FDR (false discovery rate) adjusted *p* < 0.05 was considered to be statistically significant for GO and KEGG pathway over-representation tests.

### Definition of the Stromal Score-Based Gene Signature and Prognostic Model

Univariate, LASSO, and multivariate Cox regression analyses were used to study the correlation between gene expression levels and OS of patients in training cohort. Firstly, we applied univariate CoxPH (Cox proportional hazards) regression analyses to identify genes associated with OS. Secondly, the least absolute shrinkage and selection operator (LASSO) Cox regression was used to avoid over-fitting of the model. Then, multivariate CoxPH regression analyses were used to select independent prognostic factors for OS of colon cancer patients. A multigene marker-based predictive value was calculated for each patient based on the expression level of the selected gene signature by predict.glm R function. Finally, 279 patients in TCGA cohort were divided into high-risk and low-risk groups according to the best cut-off value of predictive value. Kaplan-Meier survival curves and time-dependent Receiver operating characteristic (ROC) curve analyses were operated to evaluate the predictive efficacy of the model.

### Building and Evaluation of the Nomogram for OS Prediction of Colon Cancer

The nomogram is an effective method to predict the prognosis of patients with malignant tumors, which simplifies the complicated statistical prediction model into a readable chart to evaluate the probability of OS for individual patients (Park, 2018). Taking advantage of rms R package, in this study, we included the selected gene signature through multivariate CoxPH regression analyses together with age, gender and pathological TNM stage to build a nomogram which could predict the probability of 5-year OS for colon cancer patients. The predicted probability of the nomogram was compared with the actual probability by the calibration curve to verify the accuracy of the nomogram. A predictive line overlapping with the actual line suggests an ideal model.

### Validation of the Gene Signature in External Cohorts

To find out whether the gene signature identified from the TCGA cohort were of prognostic significance for other colon cancer cases as well, we used the datasets GSE38832 and GSE39582 from the GEO database mentioned above as external validation cohorts. Likewise, Kaplan-Meier survival analyses were applied to every single prognostic gene and the integrated model on the validation cohorts. In GSE39582, patients were divided into subgroups depending on their clinicopathological characteristics, then Kaplan-Meier survival analyses and ROC analyses were operated on each subgroup to confirm the predictive capacity of the established model.

### Statistical Analyses

Survival curves were compared using the Kaplan-Meier method and the log-rank test. DEGs were compared with Student’s *t*-tests and those with *p* < 0.05 and fold-changes larger than two were viewed as dramatically dysregulated. Clinicopathological characteristics were compared by χ^2^ tests or Wilcoxon tests. All tests were two-sided, and a *p* < 0.05 was considered to be significant unless noted otherwise. All data were analyzed using R (Version 4.0.3).

## Results

### Study Design and Brief Summary of Patients’ Information

**FIGURE 1:**
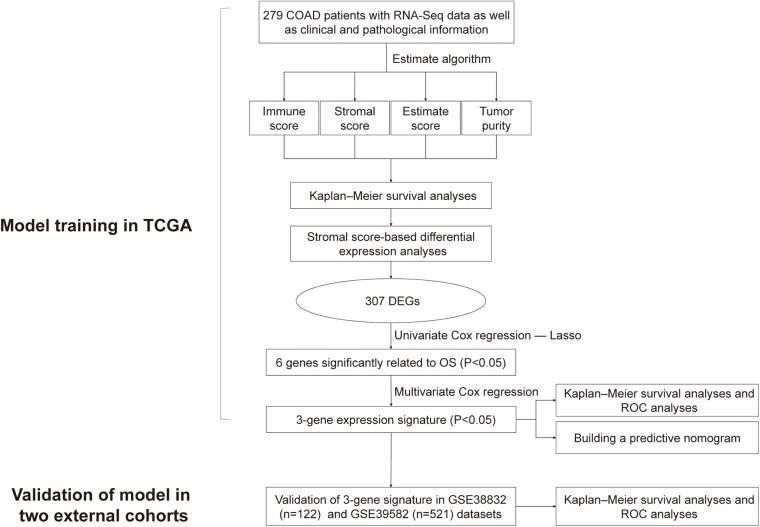
Overall flowchart of this study.

The study design is shown as a flowchart in Figure 1. In this chart, we showed the detailed construction process of the OS prediction model for colon cancer patients. Patients’ clinicopathological data were briefly summarized in Table 1.

**TABLE 1:** Clinical and pathological characteristics of COAD patients in TCGA, GSE38832, and GSE39582 cohorts.

Characteristics	Total (n)	Percentage (%)	*P*-value
**TCGA**	**279**	**100**	
Survival status			
Alive	210	75.3	–
Dead	69	24.7	
Age			
≤65	132	47.3	–
>65	147	52.7	
Gender			
Male	154	55.2	–
Female	125	44.8	
TNM stage			
I	46	16.5	–
II	111	39.8	
III	81	29.0	
IV	41	14.7	
**GSE38832**	**122**	**100**	
Survival status			
Alive	94	77.0	0.702
Dead	28	23.0	
TNM stage			
I	18	14.7	0.019
II	35	28.7	
III	39	32.0	
IV	30	24.6	
**GSE39582**	**521**	**100**	
Survival status			
Alive	357	68.5	0.045
Dead	164	31.5	
Age			
≤65	202	38.8	0.020
>65	319	61.2	
Gender			
Male	284	54.5	0.853
Female	237	45.5	
TNM stage			
I	32	6.1	0.110
II	245	47.1	
III	156	35.7	
IV	58	11.1	

*P-values were calculated compared with TCGA cohort. TCGA, GSE38832, and GSE39582 are specific cohorts. The bold values in column “Total” indicate the sample size.*

### Stromal Scores Are Significantly Associated With Overall Survival of Colon Cancer Patients in TCGA Cohort

**FIGURE 2:**
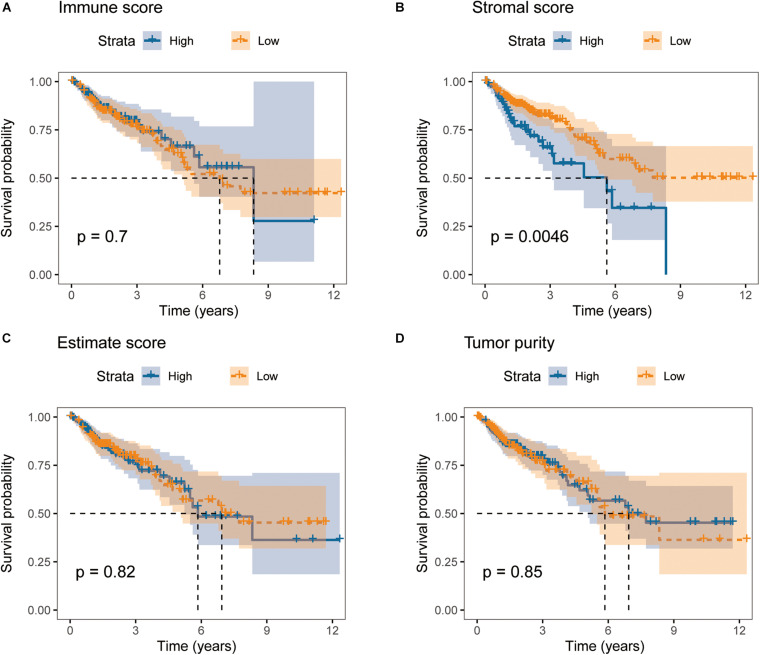
Stromal scores are significantly associated with overall survival of colon cancer patients in TCGA cohort. Kaplan-Meier curves of overall survival for patients with high vs. low **(A)** immune scores, **(B)** stromal scores, **(C)** estimate scores, and **(D)** tumor purity.

We downloaded RNA-seq data of 279 primary colon cancer patients with survival information and significant clinicopathological data from TCGA database. Based on gene expression profiles, immune scores, stromal scores, estimate scores and tumor purity were calculated for each patient, respectively, using ESTIMATE algorithm. In order to find out the potential correlation between overall survival and the four ESTIMATE scores, we divided the 279 patients into high and low groups according to their four ESTIMATE scores, respectively. Kaplan-Meier survival curves indicated that colon cancer patients with higher stromal scores showed poorer overall survival than lower ones (Figure 2B, log-rank test *p* = 0.0046). However, Kaplan-Meier survival analyses didn’t show significant differences in OS between groups with different levels of immune scores, estimate scores or tumor purity (Figures 2A,C,D).

### Comparison of Gene Expression Profiles by Stromal Scores in Colon Cancer

**FIGURE 3:**
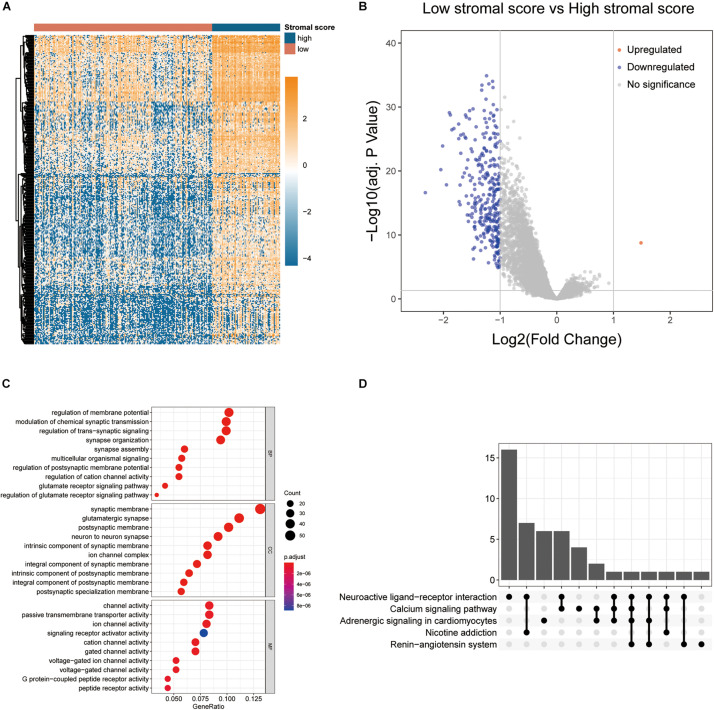
Comparison of gene expression profiles by stromal scores in colon cancer. **(A)** The heatmap showing the expression patterns of stromal score-related DEGs with unsupervised hierarchical clustering analysis. **(B)** The volcano plot visualizing the expression profiles of stromal score-related DEGs. **(C,D)** GO and KEGG enrichment analyses revealed the most significant biological process (BP), cellular component (CC), molecular function (MF), and pathways correlated to DEGs.

In order to identify the key genes contributing to the opposing survival outcomes related to stromal scores, the TCGA expression profiles of colon cancer patients with lower stromal scores were compared to those of ones with higher stromal scores. A total of 307 DEGs were identified as stromal score-related DEGs. Interestingly, among them, 306 DEGs were downregulated in patients with lower stromal scores (log2FC <−1, *p* < 0.0001) while only 1 DEG was upregulated (log2FC > 1, *p* < 0.0001). The expression profiles of stromal score-related DEGs are visualized on the heatmap (Figure 3A) and volcano plot (Figure 3B). Apparently, unsupervised hierarchical clustering analysis showed that identified DEGs could clearly distinguish patients with high and low stromal scores, and DEGs in patients with low stromal scores were mostly downregulated, which suggested that further digging might find out crucial genes responsible for poor outcomes of colon cancer patients with high stromal scores. GO and KEGG enrichment analyses showed that the DEGs mainly took part in neuro synapse assembly, biosynthesis of synapse specific membrane and function of ion channel on synaptic membrane (Figures 3C,D).

### Identification of Prognostic Gene Signature in Colon Cancer

**FIGURE 4:**
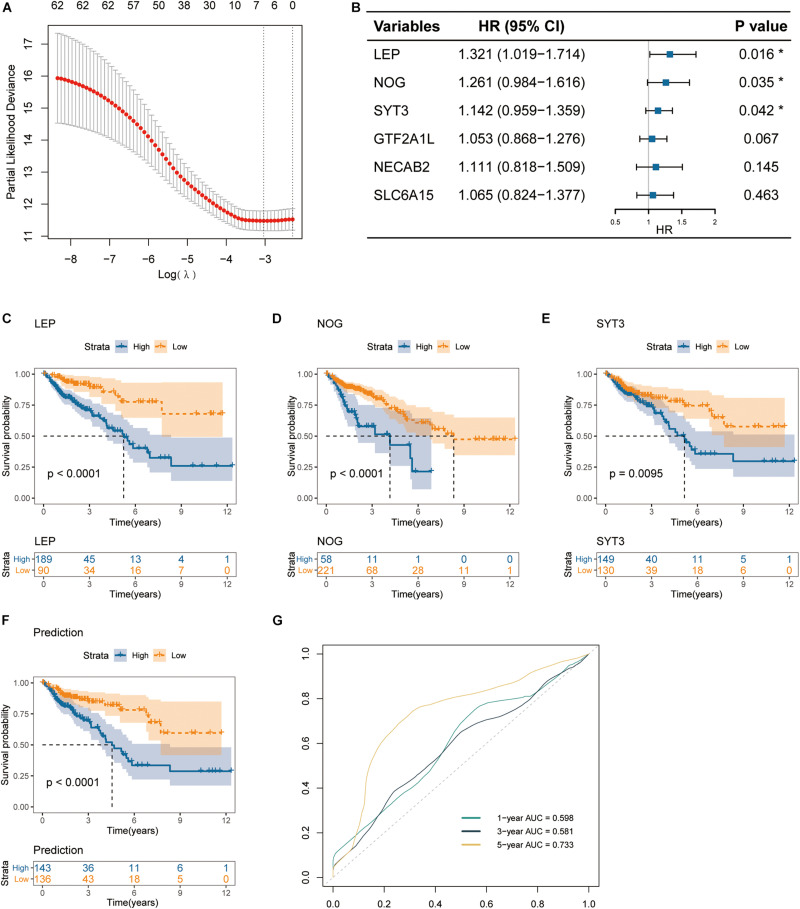
Identification of prognostic gene signature in colon cancer. **(A)** Partial likelihood deviance of different numbers of variables revealed by the LASSO regression model. **(B)** The forest plot of hazard ratios for prognostic gene signature selection applying the multivariate CoxPH regression. **(C–E)** Kaplan-Meier curves of overall survival for patients grouped by expression levels of the three signature genes: LEP, NOG and SYT3. **(F)** Kaplan-Meier curves of overall survival for the integrated prediction model encompassing the three genes. **(G)** ROC curves for 1-, 3-, and 5-year OS, with AUC values.

Still, we used the TCGA cohort as a training dataset, and the 307 DEGs above were subjected to univariate CoxPH regression analyses to identify markers that associated with OS. As a result, 64 genes with *p* < 0.05 were selected as candidates (Supplementary Material 1). Then, the LASSO regression model further identified six genes that were closely associated with OS (Figure 4A). Finally, the six genes were subjected to multivariate CoxPH regression analyses to adjust the risk scores of each selected gene for age, gender, and pathological TNM stage. A total of three genes were recognized as independent prognostic factors (*p* < 0.05), including LEP (*HR* = 1.321, 95% CI 1.019–1.714), NOG (*HR* = 1.261, 95% CI 0.984–1.616) and SYT3 (*HR* = 1.142, 95% CI 0.959–1.359) (Figure 4B). Moreover, age and TNM stage were also showed to be independent prognostic factors (Not shown).

Furthermore, the TCGA cohort was subdivided into high-expression and low-expression subgroups according to the best expression cut-off levels of the three genes [best cut-off values were calculated by Survminer R package (V.0.4.6)]. Kaplan-Meier curves revealed that high expression levels of all of the three genes were associated with inferior overall survival (log-rank test *p* < 0.0001 for LEP, *p* < 0.0001 for NOG, and *p* = 0.0095 for SYT3) (Figures 4C–E). Based on such results, we next established a multiplex prediction model encompassing the transcript expression levels of the three genes. As shown in the Kaplan-Meier curves for prediction model, patients of high risk had much worse OS rates than those of low risk (log-rank test *p* < 0.0001) (Figure 4F). ROC curves showed that the three-gene expression integrated prediction model had an area under the curve (AUC) value of 0.733 in evaluating 5-year OS (Figure 4G).

### Building of a Nomogram to Predict OS in Colon Cancer Patients

**FIGURE 5:**
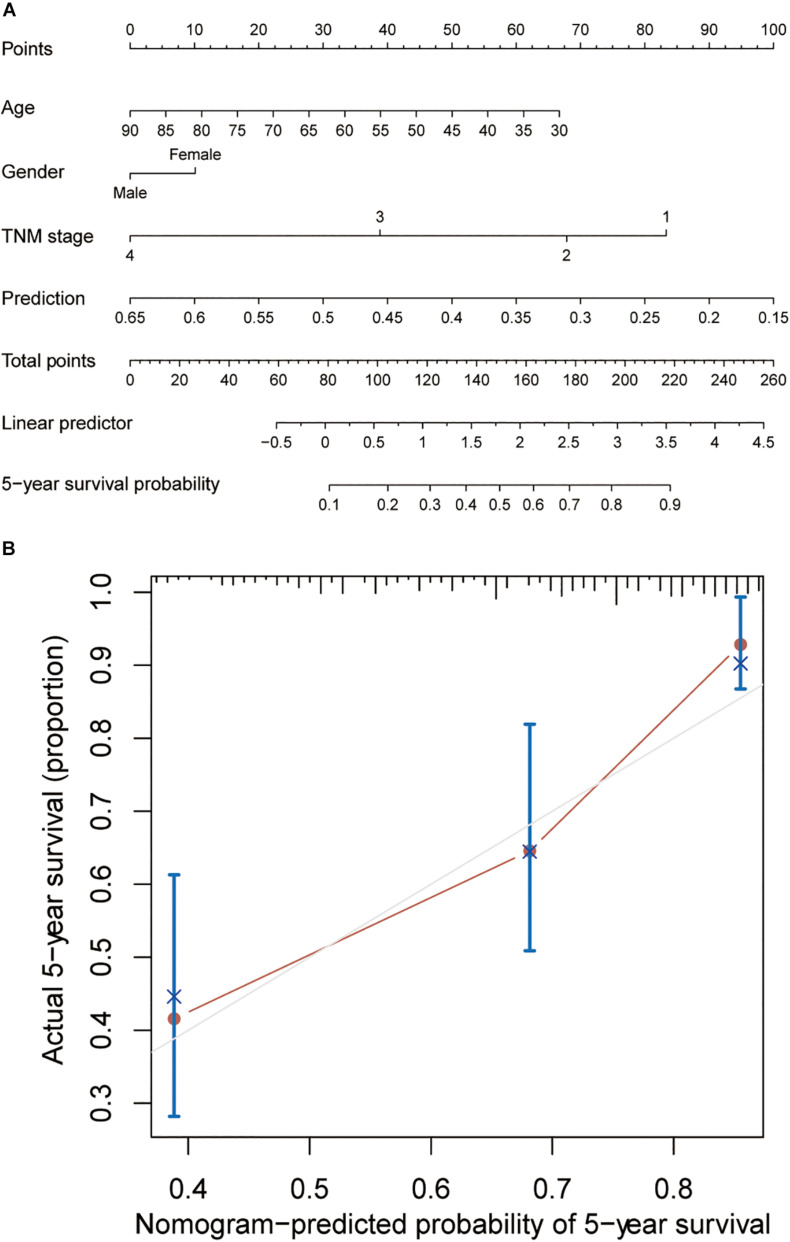
Building of a nomogram to predict OS in colon cancer patients. **(A)** The nomogram to predict 5-year overall survival probability by integrating the three-gene prediction model with age, gender and TNM stage. **(B)** The plot depicting the calibration of the nomogram in terms of the consistency between predicted and actual outcomes. Nomogram performance is shown as the plot relative to the dotted line, which represents an ideal model.

In order to establish a clinically applicable method to predict overall survival of patients with primary colon cancer, a nomogram for 5-year survival prediction was built by integrating the stromal score-associated three-gene signature, age, gender and TNM stage in TCGA cohort (Figure 5A). Further calibration plot illustrated that the nomogram performed well in comparison with the performance of an ideal model (Figure 5B), which confirmed the great predictive accuracy of the newly constructed nomogram.

### Validation of the Stromal Score-Based Gene Signature in External Cohorts

**FIGURE 6:**
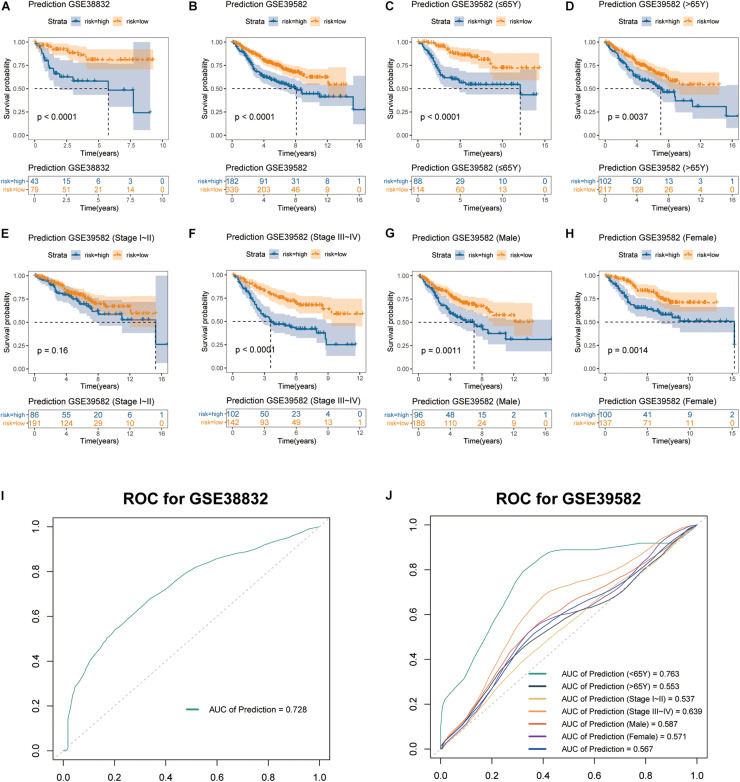
Validation of stromal score-based gene signature in two external cohorts. Kaplan-Meier curves of OS for the integrated prediction model encompassing the three genes in **(A)** GSE38832 and **(B)** GSE39582 cohorts. Subgroup analyses of OS based on **(C,D)** age, **(E,F)** TNM stage, and **(G,H)** gender of colon cancer patients in GSE39582 cohort. **(I,J)** ROC curves of the multigene prediction model for GSE38832 and GSE39582 cohorts, with AUC values.

The stromal score-based gene signature and the three-gene integrated predictive model were validated in two independent cohorts from the GEO database (GSE38832 and GSE39582). Consistent with our results obtained from TCGA cohort, each of the three genes showed to be a significant unfavorable factor to the OS of the patients in both cohorts by Kaplan-Meier survival analyses (Supplementary Material 2). Similarly, the multiplex model encompassing the three signature genes was built and patients with high risk had significantly worse OS rates than those with low risk in both cohorts (log-rank test *p* < 0.0001) (Figures 6A,B). ROC analysis showed that the AUC value of the three-gene signature model was 0.728 in evaluating 5-year OS for patients in GSE38832 cohort (Figure 6I), which was close to the result obtained from TCGA cohort (Figure 4G). Considering the heterogeneity of the cohorts, we further explored the predictive ability of the three-gene signature in different subgroups divided by age, TNM stage and gender in GSE39582. As was shown in Kaplan-Meier curves for different subgroups, the three-gene signature model could well distinguish opposing prognostic outcomes in all the subgroups except TNM stage I–II (log-rank test *p* < 0.0001 for patients ≤ 65Y, *p* = 0.0037 for patients > 65Y, *p* = 0.16 for patients of stage I–II, *p* < 0.0001 for patients of stage III–IV, *p* = 0.0011 for male patients, and *p* = 0.0014 for female patients), which suggested huger differences of OS in patients ≤ 65Y and patients of stage III–IV (Figures 6C–H). Also, ROC analyses for GSE39582 cohort were operated on the six subgroups later. To our surprise, the AUC value of the three-gene signature model in evaluating 5-year OS for patients younger than 65 showed to be a remarkable 0.763, whereas the AUC value was 0.553 for elder patients (Figure 6J). Besides, the AUC values were 0.537, 0.639, 0.587, and 0.571 for patients of stage I–II, patients of stage III–IV, male patients and female patients, respectively (Figure 6J). In this part of the study, we validated the great value of our established three-gene signature model in predicting OS for colon cancer patients, which might have especially promising prognostic value for young patients.

## Discussion

Tumor microenvironment is a crucial concept in tumor immunology, which includes immune cells, stromal cells, epithelial cells, fibroblasts, vascular cells, and signaling molecules that closely interact with the development and metastasis of tumors (Bussard et al., 2016; Meurette and Mehlen, 2018; Ren et al., 2018; Vitale et al., 2019). As the most important components of TME, immune cells and stromal cell have been deeply concerned by scientists. Currently, several gene signatures based on the characteristics of immune and stromal components in TME have been reported (Chifman et al., 2016; Charoentong et al., 2017; Hao et al., 2018; Wang et al., 2019). For colon cancer, Pages et al. (2009) built an immunoscore system based on the amounts of infiltrated CD3+, CD8+, or CD45RO+ lymphocytes in the central- and peri-tumoral areas, finding the prognostic ability of the immunoscore system stronger than TNM stage. Nonetheless, the latest multi-central clinical research showed deficiency in predictive accuracy of the system (Pages et al., 2018), probably due to the neglection of stromal components in TME. Quite recently, Zou et al. (2020) reported SNAP25 as a prognostic gene based on stromal-immune score. However, no concrete model or algorithm was built to predict survival outcomes in that study, which might largely reduce the practicability and reliability of the results. In order to fill the gap in this aspect for colon cancer, we devised the current study.

Through a specific view of the tumor microenvironment, our study was based on immune and stromal scores by means of well-established ESTIMATE algorithm. Different from the results of some of the previous studies, we found the stromal score to be the only factor significantly related to survival outcomes of colon cancer patients among the four output values of the algorithm. For this reason, we next mined for prognostic genes based on the stromal score rather than the immune score. Through a series of successive, organized and targeted analyses for transcriptomic data and survival information, this study identified a set of stromal score-related prognostic DEGs and built a stromal score-based multigene prognostic prediction model for colon cancer, which was demonstrated to be highly efficient by ROC curves. Additionally, it was interesting to suggest in our validation section that, to some extent, the established prediction model might be much more accurate for young patients. Considering the increasing mortality among young patients with colon cancer (Schoen et al., 2012), and that prognosis of colon cancer among young patients is not well known so that it is difficult to advise about adjuvant chemotherapy (Kneuertz et al., 2015; Manjelievskaia et al., 2017), our results might be of additional value for prognostic prediction and treatment decision among young patients.

All of the three signature genes identified in this study have been reported to play vital roles in tumorigenesis, development and metastasis of varieties of malignant tumors, including colon cancer. LEP, which encodes leptin, is well-known because of its significant role in obesity. Besides energy homeostasis, recent studies have shown its extended properties involving numerous aspects including the high risk of colon cancer (Stattin et al., 2004; Tamakoshi et al., 2005). Slattery et al. (2008) suggested that different leptin and leptin receptor genotypes might influence the risk of colon cancer. Although the synaptotagmins (SYTs) have not been well studied in malignant tumors yet, their roles in vesicle trafficking and fusion of vesicles have been pointed out (Chapman, 2002), which might be associated with cell migration. Particularly, Masztalerz et al. (2007) reported that SYT3 was essential for migration of T cells, which might indirectly take part in the tumor microenvironment. As an antagonist of bone morphogenetic proteins (BMP), noggin encoded by gene NOG plays a role in both normal development and cancers. It was lately reported that noggin was responsible for poor prognosis of gastric cancer by promoting the proliferation of tumor cells via upregulating EGFR (epidermal growth factor receptor) (Sun et al., 2020). In colon, Hardwick et al. (2004) found noggin inhibited apoptosis and proliferation in mouse colonic epithelium *in vivo*. Mostly consistent with the results of the previous studies, high expression levels of the three signature genes we identified all represent poor survival outcomes of patients with colon cancer.

Amounts of investigations have been done to identify key targets that play crucial roles in tumorigenesis and cancer development, some of them even explored the detailed inner mechanisms. However, it is necessary to transfer the preclinical medical findings into clinical applications. A recent study utilizing the profiles of clinical samples and TCGA cases established a prognostic prediction model of high quality for head and neck squamous cell carcinoma by encompassing both immune-related gene signature and clinicopathological factor, providing a powerful tool for the prognostic prediction in clinical practice in addition to TNM staging (Yao et al., 2020). Meanwhile, with the rapid development in techniques of next-generation sequencing, it may become more and more convenient and popularized to help evaluate prognostic outcomes and make treatment decisions for cancer patients based on transcriptomic profiles of target genes in their tumor tissues. Therefore, if correctly applied in clinical practice, the multigene prediction model constructed in our present study might have potential value for clinical management of colon cancer.

The lack of clinical specimens limited our study to some degree. Further efforts should be done to validate the model in an independent cohort of clinical samples, on both gene and protein levels. In addition, for deeper explorations, the mechanisms of the signature genes regulating tumorigenesis remain to be further investigated both *in vivo* and *in vitro*.

## Conclusion

In conclusion, we demonstrated the correlation between stromal characteristics of tumor microenvironment and survival outcomes of colon cancer patients. The novel prognostic prediction model we established based on stromal score-related gene signature might be of value to stratify patients and make clinical decisions for colon cancer.

## Data Availability Statement

Publicly available datasets were used in this study. TCGA data can be found here: https://tcga-data.nci.nih.gov/tcga/. GEO data were downloaded by GEOquery R package.

## Author Contributions

JJ, YD, and GX conceived and designed the study. JJ and YD performed the analyses. JJ, YD, QZ, PT, and QF wrote the manuscript. GX reviewed the data and manuscript. All authors read and approved the final manuscript.

## Conflict of Interest

The authors declare that the research was conducted in the absence of any commercial or financial relationships that could be construed as a potential conflict of interest.

## Footnotes

1 https://tcga-data.nci.nih.gov/tcga/
