# Supplementary material for: Stromal Score-Based Gene Signature: A Prognostic Prediction Model for Colon Cancer
Source: Front Genet. 2021 May 12;12:655855. doi: 10.3389/fgene.2021.655855 (PMC8150004; doi:10.3389/fgene.2021.655855)
Supplement: Supplementary file 1 [file Data_Sheet_1.PDF]

frontiers

# *Supplementary Material 1*

| Variables | HR (95% CI) | P value |
|---|---|---|
| APCDD1L | 1.2 (1−1.3) | 0.025 |
| LRRC4C | 1.2 (1.1−1.4) | 0.0027 |
| CCBE1 | 1.1 (1−1.3) | 0.018 |
| CRTAC1 | 1.1 (1−1.3) | 0.028 |
| LEP | 1.2 (1.1−1.3) | 0.0015 |
| FMO2 | 1.1 (1−1.3) | 0.042 |
| PCDH10 | 1.2 (1−1.5) | 0.013 |
| SLITRK4 | 1.2 (1−1.4) | 0.046 |
| THBS4 | 1.1 (1−1.2) | 0.015 |
| GDF6 | 1.2 (1−1.4) | 0.0099 |
| ADD2 | 1.2 (1−1.4) | 0.017 |
| CDO1 | 1.2 (1−1.3) | 0.028 |
| FAM43B | 1.2 (1.1−1.5) | 0.0065 |
| NLGN1 | 1.2 (1−1.4) | 0.016 |
| NRK | 1.2 (1−1.3) | 0.012 |
| KCND2 | 1.2 (1−1.3) | 0.027 |
| GAP43 | 1.1 (1−1.3) | 0.032 |
| FGF5 | 1.2 (1−1.4) | 0.022 |
| SNAP25 | 1.2 (1−1.3) | 0.014 |
| TMEM59L | 1.2 (1.1−1.4) | 0.0072 |
| GRIN2A | 1.2 (1−1.4) | 0.043 |
| PCDH9 | 1.3 (1.1−1.5) | 0.00048 |
| HAMP | 1.3 (1.1−1.5) | 0.0029 |
| MAB21L1 | 1.3 (1−1.5) | 0.026 |
| ZNF676 | 1.5 (1.2−1.9) | 0.0014 |
| SHANK1 | 1.2 (1−1.5) | 0.045 |
| SLITRK2 | 1.3 (1−1.5) | 0.018 |
| C21orf34 | 1.3 (1−1.5) | 0.017 |
| STON1−GTF2A1L | 1.2 (1−1.4) | 0.019 |
| LOXHD1 | 1.2 (1−1.5) | 0.044 |
| ZNF835 | 1.3 (1−1.6) | 0.041 |
| EXTL1 | 1.3 (1−1.5) | 0.028 |
| SYT3 | 1.3 (1.1−1.6) | 0.0033 |
| SGCG | 1.8 (1.3−2.4) | 0.00017 |
| NOG | 1.5 (1.2−1.8) | 0.00027 |
| CYP11A1 | 1.3 (1.1−1.6) | 0.0093 |
| CLEC2L | 1.3 (1.1−1.5) | 0.012 |
| SPOCK3 | 1.3 (1.1−1.5) | 0.0096 |
| ENTHD1 | 1.2 (1−1.5) | 0.041 |
| SYT6 | 1.5 (1.2−1.9) | 0.0024 |
| C2orf84 | 1.5 (1.2−1.9) | 0.0015 |
| LOC90586 | 1.3 (1−1.7) | 0.045 |
| LINGO2 | 1.3 (1−1.8) | 0.045 |
| IGSF11 | 1.2 (1−1.5) | 0.049 |
| GTF2A1L | 1.3 (1.1−1.5) | 0.0031 |
| CDR1 | 1.4 (1.1−1.9) | 0.011 |
| LOC100240735 | 1.4 (1.1−1.8) | 0.014 |
| FOXI2 | 1.4 (1.1−1.8) | 0.013 |
| SLC35F3 | 1.4 (1.1−1.8) | 0.0024 |
| PKD1L2 | 1.3 (1−1.6) | 0.045 |
| NECAB2 | 1.5 (1.2−1.8) | 0.00088 |
| SLC6A15 | 1.3 (1.1−1.4) | 0.00038 |
| ATP2B2 | 1.3 (1−1.6) | 0.044 |
| CUX2 | 1.4 (1−1.9) | 0.04 |
| UNC80 | 1.3 (1−1.7) | 0.032 |
| FAM133A | 1.3 (1.1−1.5) | 0.007 |
| HS6ST3 | 1.2 (1−1.4) | 0.018 |
| LRRC7 | 1.3 (1−1.6) | 0.026 |
| INSRR | 1.5 (1.1−2) | 0.0066 |
| CLEC4GP1 | 1.4 (1−1.8) | 0.041 |
| ZMAT4 | 1.2 (1−1.5) | 0.029 |
| WIT1 | 1.2 (1−1.3) | 0.026 |
| FOXL2 | 1.2 (1−1.5) | 0.017 |
| CGB | 1.2 (1−1.3) | 0.038 |

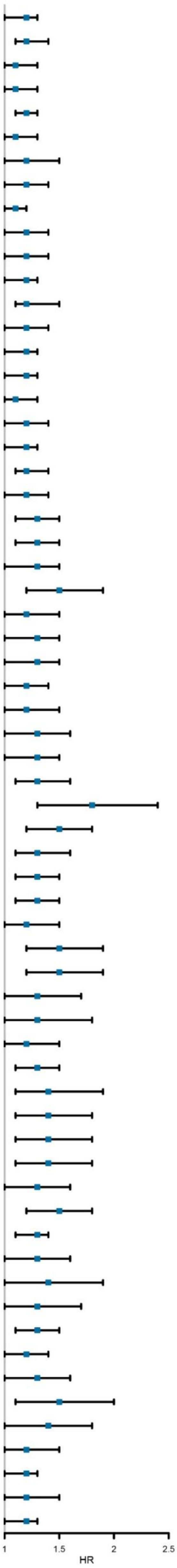


**Supplementary Figure 1.** The forest plot of hazard ratios for 64 stromal score-related prognostic DEGs. Hazard ratios and corresponding 95% confidence intervals were estimated by using the univariate CoxPH regression model.
