# Supplementary material for: Stromal Score-Based Gene Signature: A Prognostic Prediction Model for Colon Cancer
Source: Front Genet. 2021 May 12;12:655855. doi: 10.3389/fgene.2021.655855 (PMC8150004; doi:10.3389/fgene.2021.655855)
Supplement: Supplementary file 2 [file Data_Sheet_2.PDF]

Supplementary Material 2

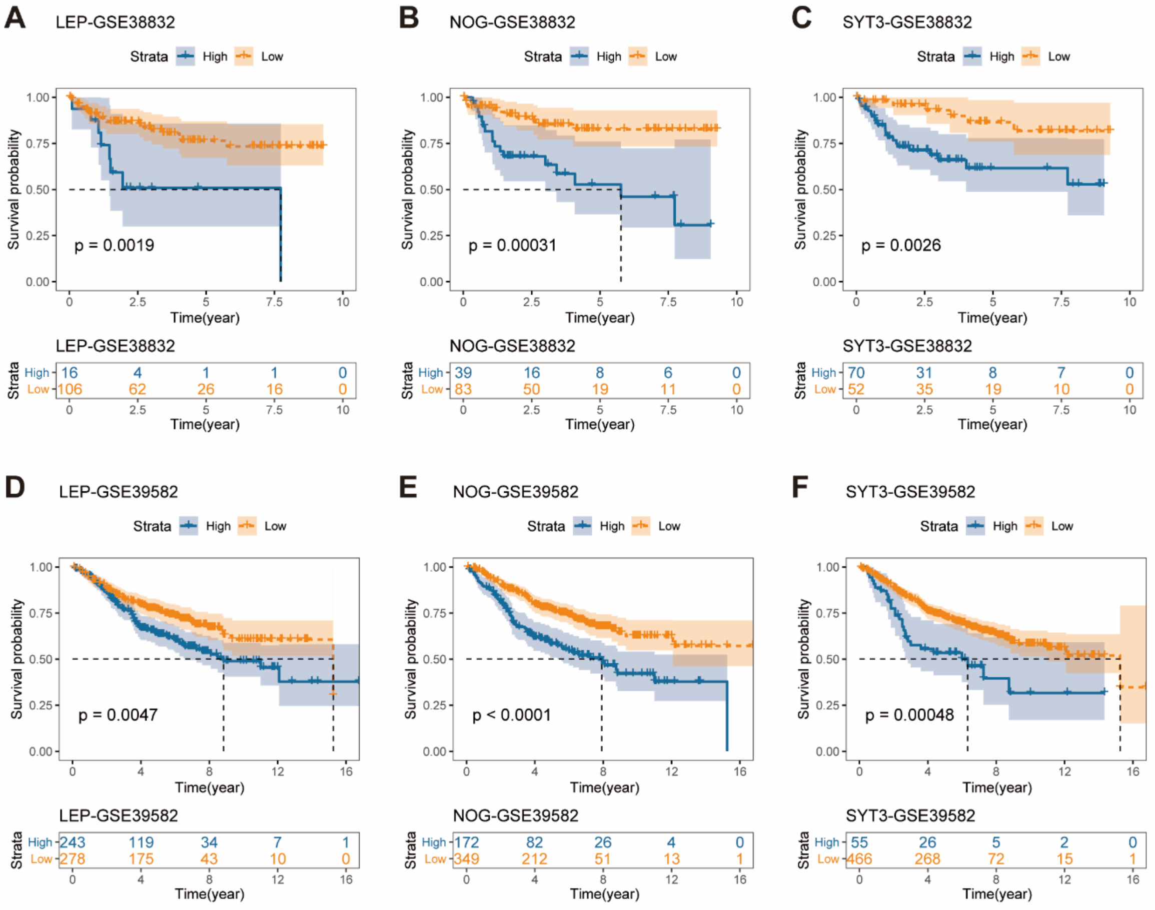

Supplementary Figure 2. Validation of the stromal score-based gene signature in GSE38832 and GSE39582 cohorts. Kaplan-Meier curves of overall survival for patients in **(A-C)** GSE38832 and **(D-F)** GSE39582 cohorts grouped by expression levels of the three signature genes: LEP, NOG and SYT3.
